# Changing clinical needs of people living with AIDS and receiving home based care in Malawi - the Bangwe Home Based Care Project 2003-2008 - a descriptive study

**DOI:** 10.1186/1471-2458-10-370

**Published:** 2010-06-24

**Authors:** Cameron Bowie, Norton Gondwe, Claire Bowie

**Affiliations:** 1 Division of Community Health, College of Medicine, Blantyre, Malawi

## Abstract

**Background:**

Home based care (HBC) has been an important component of the response to the AIDS epidemic in Africa, and particularly so before antiretroviral therapy (ART) became available. Has HBC become unnecessary now that ART is available in many African countries? One way to investigate this is to assess the changing need for comprehensive HBC as an ART programme becomes available. The Bangwe HBC programme in Malawi has been collecting data since 2003 before ART became available in 2005/6. Has the introduction of ART changed the clinical needs for HBC?

**Methods:**

Information obtained at initial assessment and follow up visits of patients receiving HBC were combined to assess case severity, survival and the response to treatment. This information was used to assess trends in mortality and the incidence, duration and severity of common symptoms over a six year period in a defined urban population in Malawi.

**Results:**

1266 patients, of whom 1190 were followed up and of whom 652 (55%) died, were studied. 282 (25%) patients died within two months of being first seen with an improvement between 2003-2005 and 2006-2008 of reduced mortality from 28% to 20%. 341 (27%) patients were unable to care for themselves on first assessment and 675 (53%) had stage 4 AIDS disease. Most patients had a mix of symptoms at presentation. Self care increased somewhat over the six years although case severity as measured by WHO staging and nutritional status did not.

350 patients were on ART either started before or after initial assessment. There were significant barriers to accessing ART with 156 (51%) of 304 stage 3 or 4 patients first assessed in 2007 or 2008 not receiving ART.

Over the six year period new HBC cases reduced by 8% and follow up visits increased by 9% a year. Between 4 and 5 people sought HBC for the first time each week from an urban health centre catchment of 100,000, which required 37.3 follow up visits each week.

**Conclusions:**

Since the availability of ART in the local health facilities and despite strenuous efforts to persuade people to seek HIV testing and ART, in practice barriers existed and half the eligible HBC patients did not have access to ART. This is one reason why the clinical need for HBC services had not changed much. In terms of quantity of care the number of new patients seeking HBC reduced by 8% a year. In terms of content of care, while there had been a marginal increase in self care the severity of illness had not changed and the survival of a significant proportion of patients generated the need for repeat visits, which increased by 9% a year. In conclusion, although the content has changed the need for HBC has not diminished despite the availability of ART.

## Background

Home based care (HBC) of people living with AIDS is an established component of the continuum of care and support advocated by WHO and UNAIDS [1] and planned by many African countries including Malawi [2]. The pattern of health problems as the disease progresses will vary from one population to another and over time [3]. Therefore, an understanding of the incidence and duration of common illnesses of such patients helps to assess the needs of a population for home based care [4]. These needs will change as the HIV epidemic matures and as new interventions such as antiretroviral therapy (ART) are introduced [5]. While the early effect of ART on individual patients in Malawi is encouraging [6-10], the effect of an ART programme on a community rather than on individual patients is less easy to measure although preliminary data suggest it may reduce mortality [11]. But what has been the effect of the ART programme on home based care needs of a community? This research attempted to describe the changing pattern of home based care over a six year period as ART became available.

The Bangwe project is a home based care (HBC) project run by the Department of Community Health, College of Medicine, University of Malawi. While providing a standard HBC service to the people of Bangwe, a township of 88,000 people adjacent to the town of Limbe near Blantyre, the opportunity is taken to collect data on the health problems of patients, their response to treatment and their nutritional status. Antiretroviral drugs were not being used during the initial study period, but began to become available free of charge in Malawi in 2005.

The project has been collecting patient data since January 2003. It has been assessing the value of the national standard treatments for home based care as promulgated by the National AIDS Commission (NAC), and initially assessed the effect of supplementary feeding for the World Food Programme [12,13]. An ART programme free for patients was established in Malawi in 2005 following a limited ART service for patients who were able to pay for their drugs. The free service became more readily available to the Bangwe community in 2006 when the local health centre started an ART clinic. The study was thus able to assess changes in home based care needs as ART became available. Had the need for HBC reduced as people with AIDS become more ambulant on ART? Had the case mix changed? What types of patients were now seeking HBC?

The study population has been well demarcated and allows estimates of disease frequencies, severity levels of symptoms, rates of recurrences and the duration of episodes. These can be used to extrapolate for planning purposes to larger urban populations [14]. Careful follow up allowed an estimation of survival [15-17].

The main object of the research was, therefore, to describe the changing pattern of home based care needs as the HIV epidemic matured and as ART became available in a community.

## Methods

### The home based care services provided

The home based care project forms part of a College of Medicine HIV/AIDS Community project which encompasses community action concerning prevention, orphan care and HBC. The project covers 15 villages. Community volunteers are recruited and trained in basic HBC as devised by the National AIDS Commission (NAC). There are usually two HBC volunteers for each village. They identify people who may need HBC, and if requested, refer the patient to the HBC team. They accompany the member of the team to see the patient, offer to undertake support tasks if the patient has no carer, and do follow up visits to assess progress and future needs.

The team, three nurses and three HBC assistants, supported by the Department of Community Health of the College of Medicine, provides the clinical care in line with national guidelines. The nurse visits the patient and confirms that the patient has a condition requiring clinical care and is unable to get to a health facility. An initial assessment at the first visit by the nurse identifies the presenting symptoms and signs. Nurses are trained in and use WHO clinical staging. Patients are followed up usually weekly or fortnightly after initial assessment depending on the needs of patients. Follow up visits are stopped once the patient no longer needs clinical care or is able to get to the health centre. Each nurse and HBC assistant carries a standard kit containing dressings and drugs and these are dispensed to treat most of the common opportunistic infections and symptoms such as chronic diarrhoea free of charge. Micronutrients and food supplements, which are sometimes distributed by NAC, are dispensed when available to those who are malnourished.

Positive living advice is offered to each patient in their home and patients are encouraged to attend a patient support group which has about 80 members at any one time and at which positive living issues are discussed. Positive living advice in Malawi includes advice about the prevention of AIDS progression in HIV positive patients and HIV infection in their partners. The features of the comprehensive HBC include therefore opportunistic infection treatment, symptomatic treatment of chronic diarrhoea, neuropathic pain and other AIDS related symptoms, skin care, micronutrient supplementation, advice about positive living, social support through volunteers and the patient support group, prophylactic antibiotics such as Cotrimoxazole, advice about the importance of HIV testing, the value of ART and the need for ART retention and adherence, the identification and management of side effects of ART and referral for ART, TB treatment and other hospital services.

The first occasion a registered patient started ART was 15 March 2004. Initially ART was only available free from the two neighbouring districts where Medicin Sans Frontiere (MSF) was located. Only a few patients managed to travel and obtain their drugs there. The first patient to get their drugs from a Ministry of Health facility was in April 2005 at Queen Elizabeth Central Hospital, Blantyre. An ART clinic was opened in Bangwe in March 2006. By January 2007 waiting times for ART were usually less than two months.

### Study population

The study area is comprised of the nine villages in Bangwe, Mzedi and parts of Limbe East wards with a population of 56,700 estimated in the last census taken in 1998. The study area was increased in 2004 to include Naminyango ward, which together with the projected population growth gives an estimated population in 2007 of 88,000. The area is an urban township in the City of Blantyre, which has a population density of 3000 per square Km. Each township is split into villages of about 4,500 people. Half the population in Bangwe is living below the poverty line, which in 2006 was equivalent to $0.50 per person per day [18]. Antenatal HIV seroprevalence was 27% in Limbe in 2003, 2005 and 2007.

### Data collection

An initial assessment form was completed at first visit. The form included basic demographic data and clinical information. A follow up form was completed at each follow up visit. No patients were discharged. Once a year in August patients who had not been seen within three months were visited to check how they were and their date of death, date of starting ART or a date of a move out of the area was recorded as appropriate.

### Study period and sample

Data had been collected from 9 of the 15 villages since the project started in January 2003 until 31^st ^August 2008. Patients seen in the 5 1/2 years who were aged between 15 and 70 years with a presumptive diagnosis of AIDS in stages 2, 3 or 4 were included in the sample. Those patients with an initially reported negative HIV test and for whom a positive test result was not available later were excluded from the trend and survival but were included in the workload analyses and description of presenting clinical features (see additional file 1). The last date seen at follow up was assumed to be the date of death of those patients lost to follow up and where death was reported. It was assumed that for those who had no follow up visit but death was reported that death occurred one week after the initial assessment date when a follow up visit would have been made. The last visit was taken as the end event for patients lost to follow up. All patients were followed up if they had not been seen within three months and their living status confirmed. The last visit date was taken to be their end event date if their living status was unable to be confirmed.

### Data analysis

Data were entered once onto an excel spreadsheet, checked and SPSS used for statistical analysis. The Extended Mantel-Haenszel test was used to test for trends of categorical data [19]. Linear regression, Pearson's coefficient and ANOVA probability statistics were used to assess trends of continuous variables. The Kaplan-Meier method was used to analyse survival and the rank log test to assess statistical significance (SPSS 16).

The staging of HIV/AIDS was based on WHO definitions [20] as used in the WHO scaling up guidelines published in 2002 [21]. More recent definitions have been used by WHO [22]. Data required to use these were collected from December 2005 onwards. The earlier definitions were used in this analysis for comparison purposes. Patients assessed as WHO clinical stage 3 or 4 meet the criteria in Malawi for ART.

### Study ethics

This study was an audit of standard care and as such was exempt from ethics research committee approval.

## Results

### Demographic characteristics of enrolled patients

1266 patients, of whom 60% were female, received care in the 5 1/2 years of the study. The average age was 33.5 years, with females being younger with an average age of 32.2 years compared to males with an average age of 35.2 years. The mean age of each group recruited in each calendar year had increased slightly over the study period from 31.8 to 34.0 years (Table 1). The proportions of males and females had not changed over the study period.

**Table 1:** Characteristics of Bangwe home based care patients 2003-2008

Patient characteristic	Year of assessment	2003		2004		2005		2006		2007		2008		Total		Extended Mantel-Haenszel test for trend		
		
		Number	*%*	Number	*%*	Number	*%*	Number	*%*	Number	*%*	Number	*%*	Number	*%*	chi-sq.	DF	p
Number		247		174		213		290		213		129		1266				

Sex	male	102	*41.3*	75	*43.1*	94	*44.1*	105	*36.2*	81	*38.0*	51	*39.5*	508	*40.1*	1.41	1	0.24
	
	female	145	*58.7*	99	*56.9*	119	*55.9*	185	*63.8*	132	*62.0*	78	*60.5*	758	*59.9*			

Chronic fever, cough or diarrhoea	no	222	*89.9*	145	*83.3*	189	*88.7*	272	*93.8*	204	*95.8*	123	*95.3*	1155	*91.2*	15.2	1	< 0.001
	
	yes	25	*10.1*	29	*16.7*	24	*11.3*	18	*6.2*	9	*4.2*	6	*4.7*	111	*8.8*			

Bed-bound	not bedbound	85	*34.4*	55	*31.6*	54	*25.4*	104	*35.9*	106	*49.8*	54	*41.9*	458	*36.2*	28.7	1	< 0.001
	bedridden less than half the day	29	*11.7*	17	*9.8*	66	*31.0*	70	*24.1*	43	*20.2*	30	*23.3*	255	*20.1*			
	
	bedridden more than half the day	133	*53.8*	102	*58.6*	93	*43.7*	116	*40.0*	64	*30.0*	45	*34.9*	553	*43.7*			

Can leave house	yes	173	*71.2*	128	*75*	130	*61.0*	203	*72.2*	151	*72.9*	81	*68.1*	866	*70.2*	0.77	1	0.38
	
	no	70	*28.8*	43	*25*	82	*38.5*	78	*27.8*	56	*27.1*	38	*31.9*	367	*29.8*			

Needs help bathing	yes	39	*15.8*	55	*31.6*	49	*23.0*	35	*12.1*	27	*12.7*	19	*14.8*	224	*18.5*	58.3	1	< 0.001
	
	no	158	*64.0*	118	*67.8*	163	*76.5*	255	*87.9*	185	*87.3*	109	*85.2*	988	*81.5*			

Needs help dressing	yes	42	*17.0*	30	*17.2*	20	*9.4*	26	*9.0*	17	*8.0*	10	*7.8*	145	*11.5*	4.41	1	0.04
	
	no	205	*83.0*	144	*82.8*	193	*90.6*	262	*90.3*	196	*92.0*	119	*92.2*	1119	*88.4*			

Needs help eating	yes	22	*8.9*	19	*10.9*	4	*1.9*	12	*4.2*	11	*5.2*	6	*4.7*	74	*5.9*	7.27	1	0.01
	
	no	224	*90.7*	155	*89.1*	209	*98.1*	273	*95.8*	201	*94.8*	123	*95.3*	1185	*94.1*			

Needs help walking	yes	88	*35.6*	50	*28.7*	25	*11.7*	40	*13.9*	31	*14.6*	20	*15.5*	254	*20.1*	43	1	< 0.001
	
	no	159	*64.4*	124	*71.3*	187	*87.8*	247	*86.1*	181	*85.4*	109	*84.5*	1007	*79.9*			

Needs help with toileting	yes	70	*28.3*	40	*23.0*	24	*11.3*	40	*14.1*	29	*13.9*	19	*15.0*	222	*17.8*	21.4	1	< 0.001
	
	no	176	*71.3*	133	*76.4*	188	*88.3*	243	*85.9*	179	*86.1*	108	*85.0*	1027	*82.2*			

Past TB	yes	82	*34.0*	60	*35.3*	86	*42.0*	99	*34.7*	56	*27.2*	40	*31.0*	423	*34.2*	1.99	1	0.16
	
	no	159	*66.0*	110	*64.7*	119	*58.0*	186	*65.3*	150	*72.8*	89	*69.0*	813	*65.8*			

On TB treatment at assessment	yes - pulmonary	75	*30.4*	58	*33.3*	73	*34.3*	63	*21.7*	36	*17.0*	24	*18.6*	329	*26.0*	13.28	1	< 0.001
	
	yes - extra-pulmonary	2	*0.8*	15	*8.6*	12	*5.6*	10	*3.4*	7	*3.3*	10	*7.8*	56	*4.4*			
	
	no	170	*68.8*	101	*58.0*	128	*60.1*	217	*74.8*	169	*79.7*	95	*73.6*	880	*69.5*			

HIV test done before assessment	yes	47	*19.0*	72	*41.4*	97	*45.5*	186	*64.1*	154	*72.3*	96	*74.4*	652	*51.5*	185	1	< 0.001
	
	no	200	*81.0*	102	*58.6*	116	*54.5*	104	*35.9*	59	*27.7*	33	*25.6*	614	*48.5*			

ART status	on ART before initial assessment	0	*0.0*	0	*0*	0	*0.0*	35	*12.1*	75	*35.2*	48	*37.2*	158	*12.5*	207	1	< 0.001
	
	on ART after initial assessment	19	*7.7*	28	*16.1*	37	*17.4*	66	*22.8*	28	*13.1*	14	*10.9*	192	*15.2*			
	
	not on ART	228	*92.3*	146	*83.9*	176	*82.6*	189	*65.2*	110	*51.6*	67	*51.9*	916	*72.4*			

WHO staging	2	30	*12.1*	17	*9.8*	15	*7.0*	21	*7.2*	30	*14.2*	18	*14.0*	131	*10.3*	2.84	1	0.092
	
	3	82	*33.2*	49	*28.2*	89	*41.8*	114	*39.3*	80	*37.7*	45	*34.9*	459	*36.3*			
	
	4	135	*54.7*	108	*62.1*	109	*51.2*	155	*53.4*	102	*48.1*	66	*51.2*	675	*53.3*			

Sputum sent for AFB	yes	0	*0*	0	*0*	18	*8.5*	26	*9.2*	14	*6.6*	6	*4.7*	64	*5.1*	15.7	1	< 0.001
	
	no	247	*100*	174	*100*	195	*91.5*	263	*92.9*	199	*93.4*	122	*95.3*	1200	*94.9*			

Referred for ART	yes	0	*0*	0	*0*	12	*5.6*	39	*13.5*	36	*16.9*	23	*17.8*	110	*8.7*	76.8	1	< 0.001
	
	no	247	*100*	174	*100*	201	*94.4*	250	*86.5*	177	*83.1*	106	*82.2*	1155	*91.2*			

Died, alive or lost to follow up	died	171	*69.2*	126	*72.4*	131	*61.5*	129	*44.5*	68	*31.9*	27	*20.9*	652	*51.5*	153	1	< 0.001
	
	lost	8	*3.2*	9	*5.2*	29	*13.6*	27	*9.3*	19	*8.9*	3	*2.3*	95	*7.5*			
	
	only seen once	23	*9.3*	4	*2.3*	6	*2.8*	13	*4.5*	8	*3.8*	22	*17.1*	76	*6.0*			
	
	alive	45	*18.2*	35	*20.1*	47	*22.1*	121	*41.7*	118	*55.4*	77	*59.7*	443	*35.0*			

		**Rate**		**Rate**		**Rate**		**Rate**		**Rate**		**Rate**		**Rate**				

First visit rate per 1000 population		4.2		2.5		2.7		3.5		2.5		2.2		2.9		31.2	1	< 0.001

Follow up visit rate per 1000 pop		10.7		18.1		23.6		27.0		21.4		17.6		20.1		84.2	1	< 0.001

		**Mean**	***SD***	**Mean**	***SD***	**Mean**	***SD***	**Mean**	***SD***	**Mean**	***SD***	**Mean**	***SD***	**Mean**	***SD***	**Pearson r**	**Anova p**	

Body mass index		18.6	*3.2*	17.9	*2.3*	18.1	*2.7*	18.3	*3.2*	18.3	*2.9*	18.0	*3.3*	18.3	*3.0*	-0.022	0.47	

Age		31.8	*7.9*	33.0	*8.0*	32.8	*8.1*	34.8	*9.0*	34.4	*8.1*	34.0	*9.2*	33.5	*8.4*	0.11	< 0.001	

A slight majority of patients (54%) were married, a third (35%) were either divorced or widowed and 10% single, which for the latter two groups may compromise household caring capacity. The majority of patients in Bangwe were Christian with 11% Muslim. The average number of people in the households was 5.1.

### Stage of disease at presentation

Many patients presented in advanced stage of disease, with 675 (53%) having stage 4 disease. A slightly larger proportion of patients presented with Stage 2 disease in 2007 and 2008 than in previous years. However there was no change in the proportions of patients with advanced disease over the period (chi sq. 2.84; 1 degree of freedom; p = 0.092) (Table 1).

### HIV test status

HIV counselling and testing (HCT) was becoming more available and popular in Malawi. Of the home based patients seen at first assessment just over half had already been tested, changing from 22% in 2003 to 74% in 2008. The vast majority (84%) who had not had a test said they wanted to have one, increasing from 70% in 2003 to 100% in 2005 but down to 78% in 2008. HCT became available at the local health centre in 2005 and in the patient's home to those unable to get to a testing site in 2008.

### Treatment prior to assessment

TB had been treated in 34% of patients prior to being seen by the home based care team. The median time between TB diagnosis and being seen by the home based care team was 20 weeks (25% in 7 weeks and 75% in 96 weeks). Most of these patients (385 of 423) were on TB treatment at the time of assessment. 329 where treated for pulmonary and 56 for extra-pulmonary TB. There were fewer patients on TB treatment in the later half of the study period, with 31% in 2003 and 26% in 2008 (Table 1).

**Figure 1:**
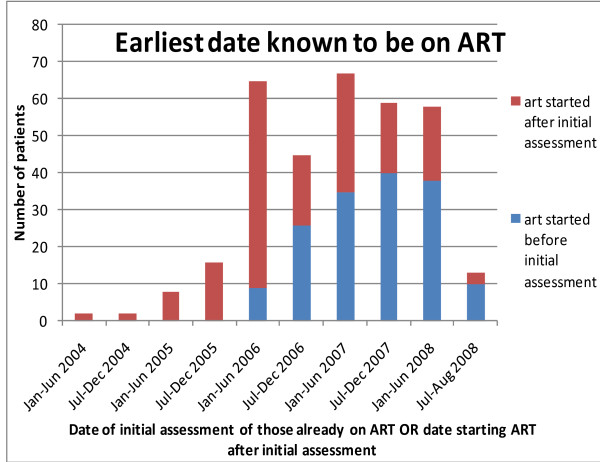
**Dates of starting ART for Bangwe patients who started ART after initial assessment AND dates of initial assessment of those already on ART - 2003-2008**.

No one was on ART at initial assessment in 2003 - 2005 (Figure 1). The first patient seen in this way was in June 2006. By 2008, 37% of patients were already on ART when first seen.

### Nutritional status

Nearly all patients (93%) thought they had lost weight. Of those who could remember their healthy weight, the average weight loss was 14 kg. The mean BMI at presentation was 18.3 kg/m^2 ^(Standard Deviation 3.0) (Table 1). Over half (58%) of the patients were malnourished with a body mass index (BMI) of less than 18.5 kg/m^2 ^on enrolment. A fifth (20%) was severely malnourished below 16 kg/m^2^. There was no change in nutritional status of patients when first seen over the six years (r = -0.022; Anova p = 0.47).

### Activities of daily living

925 (73%) of the patients at initial assessment were able to care for themselves and 367 (30%) said they were able to continue normal activities. Of the others, many had multiple needs for caring support. A fifth needed help with washing, walking or the toilet, 12% with dressing, and 6% with eating (Table 1). In the previous seven days just under half (44%) had spent more than half of each day lying down. A statistically significant trend of less dependency was found for each activity of daily living assessed through the study period. While in 2003 30% of people needed help with one such activity, by 2008 only 15% needed such help. Fewer patients were bed-bound - changing from 66% in 2003 to 58% in 2008. However, there was no change in the proportion able to leave the house (chi sq 0.77; DF 1; p = 0.38).

### Symptoms

Multiple symptoms were often present on first assessment. The common symptoms were headache, cough, shortness of breath, fever and chest pain. Details of each symptom with, for selected symptoms, the duration of symptom at first assessment, the duration of the first episode and the number of repeat episodes per year are found in additional file 1.

The proportion of patients with chronic fever, cough or diarrhoea (of more than three weeks) reduced from 10.1% in 2003 to 4.7% in 2008.

### Diagnoses

93 (7%) of patients presented with lymphadenopathy. Of these patients just over half (51) were categorised as persistent generalised [more than three node groups with two nodes of more than 1.5 cm diameter for more than one month with no obvious cause].

A number of patients presented with Kaposi's sarcoma. Data were collected specifically to identify these patients from 2005 onwards. Of this recent series of patients, 8.4% had Kaposi's sarcoma, most commonly of the leg and groin. 13 of the entire study population developed Kaposi's sarcoma after initial assessment.

Specific diagnosis required to stage AIDS using the revised WHO classification were collected from December 2005. The number of specific diagnoses identified will have been an underestimate because of the failure of hospitals to document diagnoses in the patient's health passport. The most common diagnosis was recurrent upper respiratory infection followed by severe bacterial infection and a past history of oral candidiasis (Table 2).

**Table 2:** specific AIDS staging diagnoses recorded at the time of initial assessment of Bangwe patients from December 2005 (n = 1470)

Specific HIV staging diagnoses	Number	Percent
recurrent URTI	308	42.6%
severe bacterial infection	149	20.6%
past oral candidiasis	142	19.6%
herpes simplex > 1 month	30	4.1%
oesophageal or lung candidiasis	28	3.9%
non-typhoidal salmonellosis	23	3.2%
cryptococcosis	16	2.2%
cardiomyopathy	6	0.8%
toxoplasmosis	4	0.6%
cryptosporidia	4	0.6%
HIV encephalopathy	4	0.6%
systemic mycosis	3	0.4%
PCP	2	0.3%
cancer cervix	2	0.3%
atypical mycobacteriosis	1	0.1%
acute necrotising gingivitis	1	0.1%
**Total**	**723**	

### Antiretroviral therapy

158 (12%) patients were on ART treatment at the time of initial assessment. In addition a further 192 (15%) patients received ART after initial assessment (Table 1). The proportion who received ART after initial assessment increased from 8% in the 2003 cohort to a high of 23% in the 2006 cohort reducing to 11% in 2008. However, the vast majority of patients (72%) did not receive ART. Even in 2007 and 2008 131 patients out of 271 who were Stage 3 or 4 had not received ART. The barriers to receiving ART for these 131 patients included being too sick to get to an ART clinic and early death (27), a refusal to have a HIV test (53), on TB treatment (29) and other reasons (22) such as difficulty getting to an ART clinic or deciding not to start ART after counselling or being seen in an assessment clinic.

### Survival

**Figure 2:**
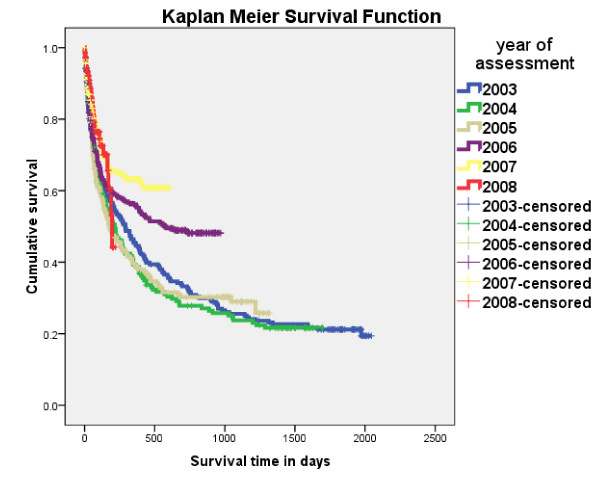
**Survival of Bangwe home based care patients 2003-2008 by year of presentation**.

**Figure 3:**
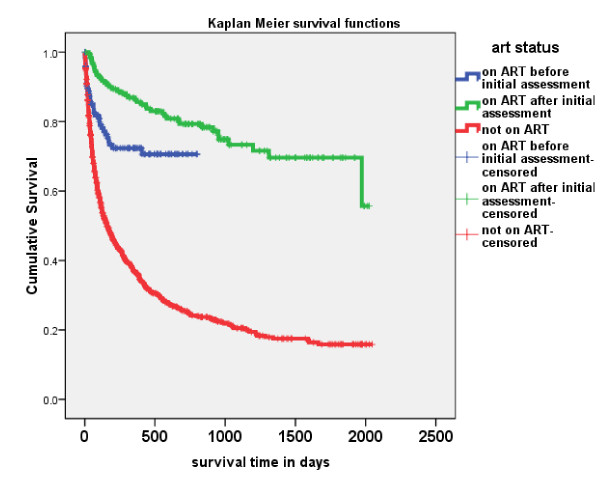
**Survival of Bangwe home based care patients 2003-2008 on or not on ART**.

Of the 1190 patients followed up, 652 (55%) died. 282 patients (25%) died within 2 months of being first seen and 403 (36%) died within the first four months. There was an improvement in 2 month mortality between the early period (2003-2005) and the late period (2006-2008) reducing from 28% to 20%. Longer term survival of more than six months did not change much between 2003 and 2005 but improved in 2006 and 2007 (Figure 2). Those on ART after HBC had a much improved survival, with 94% of patients surviving over 6 months, but those who were on ART at initial assessment had a high early mortality (Figure 3). Of the 852 who had been followed up and who had not received ART 574 (67%) died leaving 278 (33%) alive at the time of their last follow up. Of these 852 cases 37% survived one year, 25% two years, 21% three years, 18% four years and 16% five years (Table 3). For those eligible for ART (stage 3 and 4 patients) 34% survived one year, 23% two years, 19% three years, 16% four years and 15% five years. For these patients the differences of survival between last known reported HIV positive and unknown status were not statistically different (LogRank Chi-sq 1.32, 1df, p = 0.251).

**Table 3:** Survival of patients followed up who never had ART by their staging at initial assessment - Bangwe 2003-2008

Cumulative survival - Kaplan Meier survival function of all followed up patients not on ART by HIV known status												
		At initial assessment	survive 1 yr		survive 2 yr		survive 3 yr		survive 4 yr		survive 5 yr	
Last known HIV status	WHO stage	number	percent	number known alive	percent	number known alive	percent	number known alive	percent	number known alive	percent	number known alive
HIV Positive	Stage 2	32	80	8	54	3	54	1	54	1	54	1
	Stage 3	131	36	32	25	15	20	5	20	2	0	0
	Stage 4	202	28	43	20	22	15	8	12	3	0	0
	**Stages 3 + 4**	**291**	**30**	**60**	**22**	**27**	**18**	**9**	**15**	**4**	**0**	**0**
	All cases	365	34	83	24	40	19	14	17	6	13	1
HIV not known	Stage 2	55	57	24	43	12	31	8	27	5	22	3
	Stage 3	169	37	46	26	27	22	17	17	9	15	5
	Stage 4	263	35	74	23	38	20	24	17	16	17	10
	**Stages 3 + 4**	**432**	**36**	**120**	**24**	**65**	**21**	**41**	**17**	**25**	**16**	**15**
	All cases	487	37	144	26	77	22	49	18	30	17	18
All cases	Stage 2	87	64	32	47	15	36	9	32	6	26	4
	Stage 3	300	37	78	26	42	21	22	17	11	14	5
	Stage 4	465	32	117	22	60	18	32	15	19	15	10
	**Stages 3 + 4**	**765**	**34**	**195**	**23**	**102**	**19**	**54**	**16**	**30**	**15**	**15**
	All cases	852	37	227	25	117	21	63	18	36	16	19

Test of equality of survival distributions for the different levels of the last known HIV status adjusted for staging using Log Rank (Mantel-Cox) test is Chi-sq 1.32 with 1 degree of freedom and p = 0.251

### Home based care team workload

**Figure 4:**
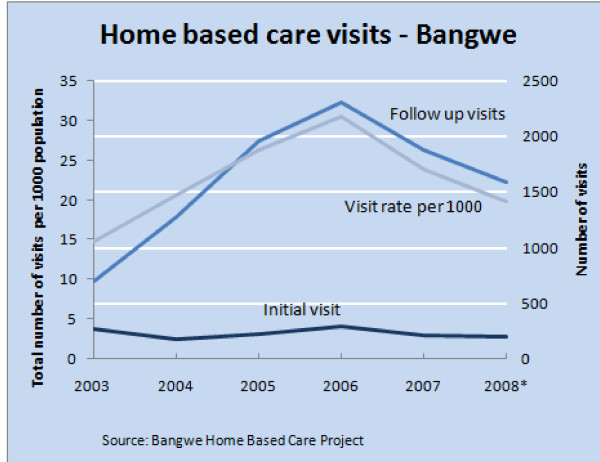
**Home based care visits from 2003 to 2008 in Bangwe**.

Referrals to the HBC team reduced over the 6 years by an average of 8.2% (95% Confidence Intervals of -17% and +2%) per year from 4.2 to 2.5 new visits per 1000 population (Table 1). Follow up visits increased by an average of 9.3% (CI of -7% and + 28%) from 14.8 to 19.8 visits per 1000 population (Figure 4).

For the 1326 patients initially assessed, a fifth (21%) was not followed up, either because the patient died, was admitted to hospital or moved away. The average number of follow up visits per person was 6.8. The average intensity of visits was 3.9 per year, with a few severely ill patients receiving intensive daily visits usually immediately prior to death.

For workload planning purposes, for a total population (all ages) of 1000 persons, taking the average workload for 2007 and the first eight months of 2008 there were 2.4 new patients and 19.4 follow up visits a year, equivalent to 4.6 new patients and 37.3 follow up visits a week.

## Discussion

The main finding of the study is that despite the advent of ART, the number and clinical needs of HBC patients had not changed as much as might have been expected. There were perhaps two reasons for this. Firstly, although ART became available in Bangwe it was not easily accessible to many sick people. Half the HBC patients with WHO stage 3 or 4 were not on ART. The two common barriers to access when ART did become available were that they were either too sick or refused to be HIV tested. Secondly, of those patients on ART prior to HBC, many were very sick on presentation and received palliative care; a quarter did not survive.

For those less sick the role of the home based care has changed to include efforts to persuade eligible patients to have HCT, diagnose and treat TB, and seek ART. Home based care staff and our volunteers are in a good position to do this as they are usually known to the patient, the family and community and have built up trust over time. Because some patients were still resistant to HCT, some of our staff have been trained in HCT and now provide it in the home if appropriate. The other addition to home based care with the advent of ART was home treatment of ART, particularly dealing with side effects, treatment failure and efforts to improve compliance. Our staff were trained in this and have a good referral system both ways with the local health centre and hospital ART clinics. Following national policy they are not able to prescribe ART. For patients who are house bound compliance could be improved if our staff were able to prescribe on going treatment in patients established on ART.

There may be a certain amount of substitution of clinical care which should but is not provided at ART clinics. If these clinics fail to provide treatments for HIV related problems such as chronic diarrhoea, skin infections and other opportunistic infections but restrict their services to prescribing and dispensing ART then known HBC patients, although ambulant, will tend to seek care from the HBC team who are known by the patients to have appropriate drugs and skills. Efforts need to be made, as we have, to reduce this inappropriate substitution by helping to improve the quality and range of ART clinic care.

There was a change over the six years in some but not all of the characteristics of the HBC patients when first seen. They became slightly older, less bed-bound and in need of less home care. Chronic symptoms of fever, cough and diarrhoea were less common, as was the proportion on TB treatment. Many more had had an HIV test and many more were on ART. But WHO staging found at initial assessment was no different over the 6 years. Features which had not changed were nutritional status, the higher proportion of females and being housebound.

Survival did not improve until 2006 and the effect of ART was most pronounced in those who had been seen by the team before treatment started, perhaps due to stabilisation by home based care treatment. Survival of a quarter of Stage 3 and 4 patients, some for over five years, who had never received ART, is surprising. The reason could be due to misdiagnosis in patients without known positive HIV serology. However, the analysis specifically excluded those patients who had reported a HIV negative result at initial diagnosis and for whom we had no evidence of seroconversion later. The survival patterns of patients with a known HIV positive result and of patients with no known test result were similar. Misdiagnosis is therefore unlikely. Most patients in the early years of the study were unwilling to have an HIV test and these early cases were almost certainly HIV positive. An alternative possibility for the long survival of these patients therefore is that treatment of opportunistic infections and the other features of comprehensive home based care by the team allowed the immune system to recover and the patients to revert to Stage 2 disease, a stage in which they remained, in some instances, for some years. This issue warrants further investigation.

The work load figures give an indication for health service planners of the scale and need for home based care services. While the new patient referral rate was down by 8% a year, follow up visits were up by 9%. For an urban population of 100,000 with an antenatal HIV sero-prevalence of 27% home based care is sought for the first time by between 4 and 5 people each week who together with previously assessed patients generate 37 follow up visits each week. The reason for the increase in follow up visits is thought to be twofold - more patients surviving and more suffering the results of failed treatment or side effects severe enough to require home care. Indeed the number of patients with opportunistic infections has not diminished.

## Conclusions

In conclusion, the clinical need for HBC services has not changed as much as anticipated despite the advent of ART. In terms of quantity of care, in a community where access to ART is still limited to half the eligible individuals, the number of new patients seeking HBC has reduced by less than 10% a year. In terms of quality of care, while there has been a marginal increase in self care the severity of illness has not changed and the survival of a significant proportion of patients generates the need for repeat visits for the care of these patients. The expertise of HBC staff has expanded. Those patients eligible but not availing themselves of ART need to be exhorted to do so and home management is important for those on ART who are housebound. Overall the need for HBC has not diminished despite the availability of ART in an urban setting where HIV prevalence is high.

## Competing interests

The authors declare that they have no competing interests.

## Authors' contributions

CB conceived the survey and analysed and wrote the first draft. NG and MCB undertook and supervised the data collection. All contributed to and approved the final report.

## Pre-publication history

The pre-publication history for this paper can be accessed here:

http://www.biomedcentral.com/1471-2458/10/370/prepub

## Supplementary Material

Additional file 1 **The number, frequency duration, severity of presenting symptoms of patients at initial assessment and follow up by the home based care service - Bangwe, Malawi - 2003-2008**. A table describes the number and frequency of presenting symptoms of the 1326 patients in the study. It shows the duration of the common presenting symptoms prior to initial presentation such as cough, fever, chest pain and diarrhoea and how long symptoms persisted after treatment dispensed at initial assessment. The table also describes the number and frequency of new episodes of symptoms found at follow up visits. Click here for file
