# Supplementary material for: Changing clinical needs of people living with AIDS and receiving home based care in Malawi - the Bangwe Home Based Care Project 2003-2008 - a descriptive study
Source: BMC Public Health. 2010 Jun 24;10:370. doi: 10.1186/1471-2458-10-370 (PMC2909166; doi:10.1186/1471-2458-10-370)
Supplement: Additional file 1 — The number, frequency duration, severity of presenting symptoms of patients at initial assessment and follow up by the home based care service - Bangwe, Malawi - 2003-2008. A table describes the number and frequency of presenting symptoms of the 1326 patients in the study. It shows the duration of the common presenting symptoms prior to initial presentation such as cough, fever, chest pain and diarrhoea and how long symptoms persisted after treatment dispensed at initial assessment. The table also describes the number and frequency of new episodes of symptoms found at follow up visits. [file 1471-2458-10-370-S1.DOC]

## Appendix

## The number, frequency duration, severity of presenting symptoms of patients at initial assessment and follow up by the home based care service

| **Symptom on first presentation** | **Number and Percentage of patients with symptom on initial assessment (n=1326)** | | **Duration of symptoms prior to presentation in weeks at initial assessment** | | | **Number and persistence of symptom after treatment dispensed at time of initial assessment in days^b^** | | | | **Number and percentage of patients with new episode of symptom on follow up** | | **Frequency of recurring symptom per year^b^** | | |
| --- | --- | --- | --- | --- | --- | --- | --- | --- | --- | --- | --- | --- | --- | --- |
|  | number | % | median | 75 percentile | 90 percentile | number | median | 75 percentile | 90 percentile | number | % | median | 75 percentile | 90 percentile |
| Headache | 886 | 67% |  |  |  | 293 | 21 | 63 | 154 | 241 | 18% | 1.0 | 2.1 | 4.4 |
| Cough | 844 | 64% | 4 | 16 | 32 | 352 | 21 | 49 | 112 | 818 | 62% | 0.9 | 2.4 | 5.7 |
| Shortness of breath | 841 | 63% |  |  |  | 347 | 21 | 49 | 112 | 404 | 30% | 1.2 | 2.4 | 5.1 |
| Fever in last week | 817 | 62% | 4 | 12 | 24 | 210 | 14 | 35 | 95 | 223 | 17% | 1.1 | 2.9 | 5.8 |
| Chest pain | 813 | 61% | 1 | 4 | 16 | 262 | 20 | 42 | 105 | 211 | 16% | 1.2 | 2.3 | 5.4 |
| SOB walking | 805 | 61% |  |  |  |  |  |  |  |  |  |  |  |  |
| Other pain | 752 | 57% |  |  |  |  |  |  |  |  |  |  |  |  |
| Lower limb pain | 675 | 51% |  |  |  | 310 | 28 | 88 | 244 | 193 | 15% | 1.8 | 3.0 | 26.1 |
| Poor appetite | 624 | 47% |  |  |  | 136 | 14 | 21 | 61 | 157 | 12% |  |  |  |
| Nausea or vomiting | 483 | 36% |  |  |  | 95 | 14 | 29 | 69 | 88 | 7% | 1.0 | 3.2 | 7.8 |
| Skin problems | 478 | 36% |  |  |  |  |  |  |  |  |  |  |  |  |
| Diarrhoea | 413 | 31% | 4 | 16 | 32 | 87 | 14 | 28 | 71 | 104 | 8% | 1.0 | 2.1 | 4.4 |
| Problem swallowing | 370 | 28% |  |  |  | 64 | 14 | 28 | 60 | 69 | 5% |  |  |  |
| SOB doing nothing | 365 | 28% |  |  |  |  |  |  |  |  |  |  |  |  |
| Itchy rash | 363 | 27% |  |  |  | 127 | 24 | 56 | 119 | 162 | 12% | 1.8 | 3.2 | 4.5 |
| Stomach pains | 347 | 26% |  |  |  | 101 | 28 | 61 | 160 | 73 | 6% |  |  |  |
| Previous shingles | 341 | 26% |  |  |  |  |  |  |  |  |  |  |  |  |
| Thrush | 257 | 19% |  |  |  | 44 | 14 | 21 | 42 | 32 | 2% | 1.8 | 3.2 | 4.5 |
| Urine problems | 241 | 18% |  |  |  | 40 | 15 | 35 | 259 | 28 | 2% |  |  |  |
| Back pain | 203 | 15% |  |  |  | 26 | 33 | 72 | 154 | 48 | 4% |  |  |  |
| Mouth ulcers | 165 | 12% |  |  |  | 19 | 21 | 42 | 175 | 21 | 2% | 2.0 | 3.4 | 5.9 |
| Upper limb pain | 157 | 12% |  |  |  | 36 | 21 | 54 | 137 | 102 | 8% |  |  |  |
| Urethral discharge | 115 | 9% |  |  |  |  |  |  |  |  |  |  |  |  |
| Genital ulcers | 104 | 8% |  |  |  |  |  |  |  |  |  |  |  |  |
| Blood in stools | 86 | 6% |  |  |  | 6 | 64 | 184 | 231 | 77 | 6% |  |  |  |
| Constipation | 83 | 6% |  |  |  | 15 | 14 | 28 | 139 | 5 | 0% |  |  |  |
| Haemoptysis | 81 | 6% |  |  |  |  |  |  |  | 2 | 0% |  |  |  |
| Neck pain | 79 | 6% |  |  |  | 19 | 15 | 28 | 63 | 12 | 1% |  |  |  |
| Bed sores/open skin lesions | 65 | 5% |  |  |  | 13 | 28 | 77 | 136 | 4 | 0% |  |  |  |
| Haematuria | 37 | 3% |  |  |  |  |  |  |  | 1 | 0% |  |  |  |
| Average | 396 | 30% |  |  |  | 124 | 22 | 52 | 128 | 134 | 10% | 1.3 | 2.7 | 7.2 |

b=number of patients with symptoms present on initial assessment and still present at follow up visit if follow up visit took place within 4 weeks of initial assessment
